# Practices and attitudes of bariatric surgeons in Israel during the first phase of the COVID-19 pandemic

**DOI:** 10.1186/s13584-020-00420-2

**Published:** 2020-10-30

**Authors:** Nahum Beglaibter, Orly Zelekha, Lital Keinan-Boker, Nasser Sakran, Ahmad Mahajna

**Affiliations:** 1 Department of Surgery, Hadassah Mount Scopus Medical Center, Jerusalem, Israel; 2 Israel Center for Disease Control, Ministry of Health, Jerusalem, Israel; 3 School of Public Health, University of Haifa, Haifa, Israel; 4 Department of Surgery, Haemek medical center, Afulah, Israel; 5 Department of Surgery, Rambam Medical Center, P.O. Box 9602, 31096 Haifa, Israel

**Keywords:** COVID-19, Bariatric surgery, Health policy, Elective procedures

## Abstract

**Introduction:**

Israel ranks very high globally in performing bariatric surgery (BS) per capita. In the first phase of the COVID-19 pandemic the bariatric surgeons’ community faced many concerns and challenges, especially in light of a decree issued by the Ministry of Health (MOH) on March 22nd, to ban all elective surgery in public hospitals. The aim of this study is to portray the practices and attitudes of Israeli bariatric surgeons in the first phase of the pandemic.

**Methods:**

Anonymous web-based questionnaire sent to all active bariatric surgeons in Israel. Statistical analysis was performed using SAS software package.

**Results:**

53 out of 63 (84%) active surgeons responded to the survey. 18% practice in the public sector only, 4% in the private sector only and 78% in both sectors. 76% practice BS for more than 10 years and 68% perform more than 100 procedures a year. Almost all the surgeons (98%) experienced a tremendous decrease in operations. Nevertheless, there were substantial differences by sectors. In the public sector, 86% of the surgeons ceased to operate while 14% did not comply with the government’s decree. In the public sector 69% of the surgeons were instructed by the administrators to stop operating. The majority of surgeons who continued to operate (77%) changed nothing in the indications or contra-indications for surgery. Among the surgeons who opted to refrain from operating on special sub-groups, the most frequent reasons were pulmonary disease (82%), age above 60 (64%), Ischemic heart disease (55%) and living in heavily affected communities. Roughly only half (57%) of the surgeons implemented changes in informed consent and operating room (OR) measures, contrary to guidelines and recommendations by leading professional societies. When asked about future conditions for reestablishing elective procedures, the reply frequencies were as follows: no special measures - 40%; PCR negativity - 27%; IgG positivity - 15%; waiting until the end of the pandemic- 9%.

**Conclusions:**

We showed in this nation-wide survey that the variance between surgeons, regarding present and future reactions to the COVID-19 pandemic, is high. There were substantial differences between the private and the public sectors. Although the instructions given by the MOH for the public sector were quite clear, the compliance by surgeons and administrators was far from complete. The administrators in the public sector, but more so in the private sector were ambiguous in instructing staff, leading surgeons to a more “personal non-structured” practice in the first phase of the pandemic. These facts must be considered by regulators, administrators and surgeons when planning for reestablishing elective BS or in case a second wave of the pandemic is on its way.

## Introduction

Israel is an economically developed country, small in size (22,145km^2^) and densely populated (approximately 9 million residents). The prevalence of obesity (body mass index, BMI ≥ 30 kg/m^2^) in 2014–2016 in Israel was around 22% in adults aged 18–64y [1] and 30% in elderly (65 + y old) [2], while that of morbid obesity (BMI ≥ 40 kg/m^2^) was, respectively, 1 and 2%. A total of 7500 bariatric procedures were performed in Israel during 2019. According to the International Federation for the Surgery of Obesity and Metabolic Disorders (IFSO) registry, Israel’s rate of bariatric surgeries is high, and was 835 per million in 2018 and 824 per million in 2019 [3, 4].

The COVID-19 pandemic interrupted bariatric surgery (BS) in Israel. The first cases of COVID-19 were identified by the end of February 2020. At that time, emergency regulations banned flights to and from China, and required all returning travelers from China and other Asian countries to be in home isolation for 14 days. By the beginning of March 2020 this regulation further extended to European countries and on March 12th, returning from every destination abroad required home isolation. This date also marked the application of escalating social distancing measures, such as closure of kindergartens/schools/universities, restriction of public gatherings, dramatic limitation of public transportation, closure of restaurants, shops and malls, shutting down non-essential workplaces, and banning any unnecessary outing. The number of newly diagnosed COVID-19 patients increased exponentially during the first three weeks in March; on March 19th, 244 new cases were diagnosed representing a 56% daily increase, bringing the total number of cases to 677, with 12 ventilated patients. The first victim of COVID-19 in Israel died on March 20th. The highest number of daily diagnosed cases occurred on April 2nd (*n* = 765; 6857 COVID-19 cases in total; 12.6% daily increase; 87 ventilated patients). By April 16th the number of ventilated patients reached a peak of 137 (with 12,758 cases in total, of them 257 newly diagnosed, representing a daily increase of 2.1%).

In the face of uncertainty and lack of information, decisions by surgeons, administrators and regulators were taken based on assumptions, trajectories, and clinical concerns.

With respect to elective BS during the pandemic, concerns focused mainly on three issues. The first was the fear of putting strain on limited resources like intensive care units (ICU) beds and medical personnel by performing elective procedures. The second was the fear of contaminating medical staff by patients. The third, and most worrying, was the increased risk of complications and death in morbidly obese patients undergoing surgery. Information from previous outbreaks of Influenza A (H1N1) and B showed clearly the negative impact of obesity on the contagion and the severity of the clinical course of such infections [5, 6]. Unfortunately, the data showing that obesity is an important risk factor for contagion and severity of clinical manifestations of COVID-19 [7–9] as well was not known yet at that time. Nevertheless, theoretical concerns specific to the morbidly obese population included:

- High prevalence of respiratory disorders (Restrictive Lung Disease, COPD, Sleep apnea, Asthma etc), turning morbidly obese patients more susceptible to pneumonitis and respiratory failure.
- High prevalence of Diabetes Hypertension and Ischemic Heart Disease, turning morbidly obese patients more susceptible to cardiovascular complications.
- General state of “inflammation”, turning morbidly obese patients more susceptible to ARDS and multi-organ failure.

Indeed, some members of our surgical community expressed concerns regarding BS at the very beginning of the pandemic. The then gut feeling was, that the morbidly obese population is more vulnerable perioperatively than the non-obese population hence the price of performing elective BS during the pandemic may outweigh the advantages of the procedure. On March 22nd, 2020, peak pandemic in Israel, a governmental decree banned all elective surgery (including bariatric procedures) in the public sector. No enforcement of the decree was in effect, and the Ministry of Health did not restrict elective activity of any kind in the private sector.

On April 19th, release of some social distancing and partial return to routine has started. The ban on performing elective procedures, including BS, was released on April 26th, 2020. By mid-May, Israel seems to overcome this pandemic phase, with around 16,500 COVID-19 patients in total, a mortality rate of 1.66%, and a very low increase in patients’ numbers (20 and lower daily; 0–0.2% daily increase).

The aim of this survey is to compare the practices and attitudes of bariatric surgeons in the public and the private sectors during the first phase of the pandemic in light of conflicting guidelines. Such knowledge portrays the “real world” picture and may be useful in planning future steps in preparing for a second wave of COVID-19 or future pandemics as well as concerning full return to elective surgery.

## Methods

This is a cross-sectional survey, carried out between April 22nd and May 15th 2020, by means of an anonymous, web-based questionnaire. All active bariatric surgeons in Israel (*N* = 63) received an email, which explained the study aim, and contained a link to the study questionnaire. Participants filled out the short form (around 5 min) online anonymously. The information gathered referred to bariatric surgery practice by sector (public, private) following the governmental decree dated March 22nd 2020, as well as contra-indications for bariatric procedures and use of protective equipment.

Descriptive data analysis used distributions and percentages. Correlations between certain characteristics were also studied. In order to compare practices between sectors we have created a public and a private sector groups. Both included the 38 surgeons that were employed in both sectors; the public sector group additionally included 9 surgeons that were employed solely there (*n* = 47), and the private sector group additionally included 6 surgeons employed solely there. Since one surgeon working for both sectors replied only on questions referring to the public sector, this person was not included in the private sector group (*n* = 43). Comparisons used chi-square tests. All statistical analysis was performed using SAS software package.

The survey was self-administered through the web, therefore no IRB approval was needed.

## Results

In total, all 63 bariatric surgeons were approached, and 53 of them filled out the questionnaire (84% response rate).

Most responders (46, 87%) were males, and most were aged between 45-60y (37, 70%). Over 53% had professional experience of more than 15 years, and over 43% reported that they perform 200 or more procedures annually. Only 9 (17%) and 6 (11%), respectively, reported working in the public or the private sector solely. Most (38, 72%) worked in both sectors (Table 1). No correlation was found between age and annual number of BS procedures, and the correlation between experience years and annual number of BS procedures was low (R Pearson = 0.3, *p* = 0.03).

**Table 1:** Characteristics of the study population

	N (%)
**Age group**	
35–44	3 (6)
45–60	37 (70)
> 60	13 (25)
**Gender**	
Male	46 (87)
Female	7 (13)
**Years of experience**	
1–5	4 (8)
5–10	9 (17)
10–15	11 (21)
15–20	14 (26)
> 20	15 (28)
**The number of BS you performed per year**	
0–50	5 (10)
50–100	12 (23)
100–200	13 (25)
200–300	12 (23)
> 300	11 (21)
**Working in**	
The public sector	9 (17)
The private sector	6 (11)
Both sectors	38 (72)

Table 2 describes practices of BS following the 22.3.2020 governmental decree by sector. In the public sector, 85% surgeons reported that BS were entirely stopped as opposed to 61% in the private sector (*p* = 0.006). Hospital administrations specifically issued instructions to stop BS in 72 and 7% of the public and private sector bariatric departments (*p* < 0.0001).

**Table 2:** Bariatric surgery (BS) practice following the Governmental decree (22 March 2020) by sector

	Public sector N = 47 (%)	Private sector N = 43 (%)	***P***-value (Chi square test)
**Stopped performing BS following the decree**			
Yes	40 (85)	26 (61)	**0.006**
No	7 (15)	17 (39)	
**Hospital management issued specific instructions to stop BS?**			
Yes	34 (74)	3 (8)	**< 0.0001**
No	12 (26)	36 (92)	
**Hospital management issued specific instructions to continue BS?**			
Yes	3 (6)	6 (14)	**0.2299**
No	43 (94)	36 (86)	
**Hospital management issued no specific instructions regarding BS**			
Yes	16 (35)	28 (74)	**0.0003**
No	30 (65)	11 (26)	
BS trends in hospitals continuing to perform BS			
**Had the number of BS procedures declined?**			
Yes	45 (98)	37 (86)	**0.0391**
No	1 (2)	6 (14)	
**If yes, by what rate (%)?**			
0–10	**–**	**–**	**< 0.0001**^a^
10–20	**–**	1 (3)	
20–40	3 (7)	8 (22)	
40–60	4 (9)	12 (32)	
60–70	10 (23)	2 (5)	
> 70	29 (63**)**	14 (38**)**	

^a^ Comparing decline rate of 60% or more versus decline rate lower than 60%

In both sectors most surgeons reported a decline in the number of BS performed during the pandemic (98% in the public sector; 86% in the private sector, *p* = 0.0391). A decline of 60% and over was more common in the public (85%) compared to the private (43%) sector (*p* < 0.0001).

**Fig. 1:**
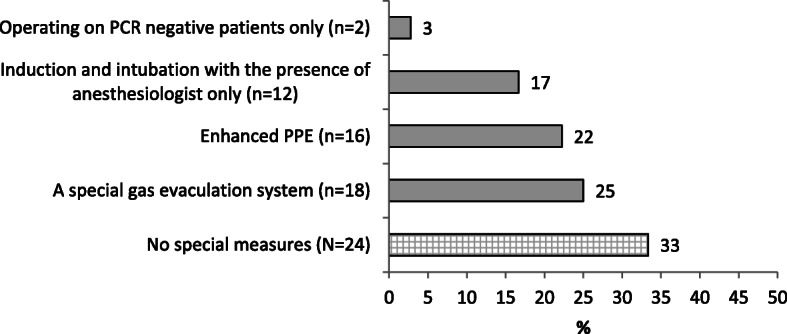
Special measures taken in the OR during the first phase of the COVID-19 pandemic. More than one option could be selected, total replies were 72. Horizontal numbers are percent of all replies received

Most surgeons (77%) reported no specific contra-indications with respect to BS performance during the pandemic. The surgeons who implemented “new” COVID-19 related contra- indications (23%) referred mostly to age (over 60y or over 70y) and background morbidity (diabetes, hypertension, ischemic heart disease, chronic lung disease) (Table 3). More than half of the surgeons (57%) reported that they informed their patients regarding a potentially increased risk of BS performance during the pandemic. The others did not think that the risk is increased (31%) or perceived the increase in risk as minimal (12%) and did not acknowledge their patients about it (Table 3). When asked about OR protection practices (multiple replies were possible), the most common response (out of 72 available answers) was that no special measures were taken (24, 33%). The next most common replies indicated a special laparoscopic Insufflated gas evacuation system (18, 25%) and enhanced personal protective equipment (16, 22%) (Fig. 1). Only 14 (28%) surgeons reported applying changes to the post-operative supervision of BS patients during the pandemic.

**Table 3:** Contra-indications and practices regarding BS during the pandemic

	All sample, N (%)
**Were there specific BS contraindications?**	
Yes	12 (23)
No	41 (77)
**If yes, did these contraindications refer to**^a^	
Patients aged > 60 years	7 (18)
Patients aged > 70 years	4 (10)
Patients with diabetes	5 (13)
Patients with hypertension	3 (8)
Patients with lung disease	8 (21)
Patients with Ischemic heart disease	5 (13)
Patients from highly infected area	7 (18)
**Did you inform your BS patients about potentially increased risk?**	
Yes	28 (57)
No, I don’t think that the risk is increased	15 (31)
No, the increase in risk is minimal	6 (12)

^a^more than one option could be selected

**Fig. 2:**
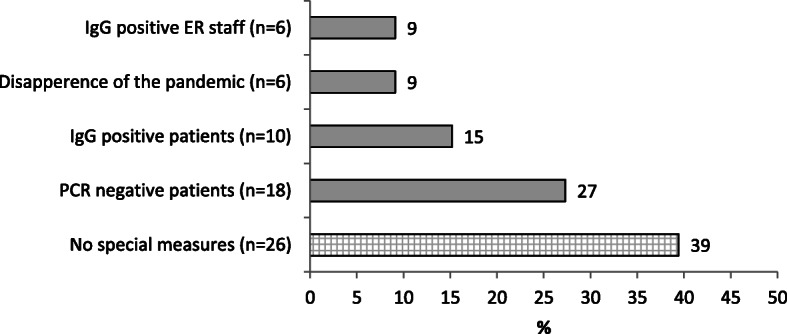
Desired conditions for resuming elective BS. More than one option could be selected, total replies were 66. Horizontal numbers are percent of all replies received

Surgeons were also asked, under what conditions regular activity should be resumed. Multiple replies were allowed and 66 were received in total. The most frequent answer was (26, 39%%) t that no special conditions were needed. The next most frequent answer (18, 27%) was that operations should only be carried out on patients that are PCR-negative. Operating only on patients that are IgG-positive to COVID-19, was the next frequent reply (10. 15%). The options of resuming elective BS only upon the end of the pandemic (6, 9%) or having only IgG positive OR staff operate (6, 9%) were less frequent (Fig. 2). Interestingly, two surgeons reported on two patients that were diagnosed with COVID-19 following BS. Because of the anonymous nature of this survey, we could not track down those patients or the surgeons who operated on them.

## Discussion

The COVID-19 pandemic posed many challenges regarding elective BS. It was well known from previous viral epidemics that obesity by itself is a risk factor for higher contagion rate, more severe clinical course and even reduced efficacy of vaccination [5, 6, 10]. At the beginning of the pandemic, data concerning COVID-19 and obesity was not available yet. Two early report one from China (191 patients) and one from Italy (1591 patients) failed even mentioning obesity as a risk factor [11, 12]. As data started to accumulate it became clear that obesity is a significant risk factor for being infected by COVID-19 and for a severe clinical course once infected [7–9].

The survey referred to the period between 22.3.2020 and 1.5.2020. Four hundred twenty three BS surgeries were performed in Israel during this period: 330 (78%) in private hospitals and 93 (22%) in public centers. In 2019, during the same calendric period, 856 bariatric surgeries were performed, 497 (58%) in a private hospitals and 359 (42%) in public hospitals. This accounts for a total reduction in elective BS of 49%. BS decrease in the private and public sectors was 32 and 73% respectively. According to our survey almost 100% of the surgeons in the public sector encountered a tremendous decline in surgical activity, 63% of them witnessed a more than 70% decline.

The “real life” practice of BS during the pandemic was a result of an interplay between three main parties, hospital administrations, the surgical community and governmental regulators (Israeli Ministry of Health). In a very simplistic way it can be said that each of those parties had different, and sometimes conflicting, interests and goals.

### Hospital administrations

In Israel the public sector hospitals are funded by governmental budget and reimbursement for procedures from four public non-governmental Health Medical Organizations (HMOs). The private sector hospitals are funded solely by reimbursement from insurance companies. The interest of a public hospital is two sided. On one hand, as a potential site for COVID 19 patients, the interest is to minimize strain on hospital resources like ICU beds and medical staff, hence, cancelation of elective activity including BS. On the other hand, the economical and operational strength of the public hospitals depends heavily on monetary income from the HMOs, hence, the interest of performing at least part of the elective activity. Indeed, our survey demonstrates a clear difference in behavior between the public and the private sectors, although the biology of COVID 19 was probably the same in both sectors. While hospital administrations specifically issued instructions to stop BS in 72% in the public sector only 7% of the private sector administrations acted in the same manner (*p* < 0.0001).

### Ministry of Health

As outlined in the introduction the Israeli government adopted at the first phase of the pandemic a strict policy of isolation, social distancing, and even lockdown. Obviously, the goal of the regulator regarding hospitals activity, was to mitigate the effect of the pandemic, to postpone reaching the limit of resources inside hospitals and to avoid collapse of the public health care systems. These goals, in the acute phase of the pandemic, paid little attention to the future economic health of public hospitals. In a decree by the Ministry of Health all elective non-cancer surgeries were banned since the 22nd of March 2020, but only in **public** hospitals. This, and the fact that the decree was never enforced on public hospital opting continuing to perform elective bariatric surgery, may give the impression that obese patients and staff safety were not considered by themselves a reason to stop elective surgery.

### The surgical community

As early as the beginning of March 2020 some bariatric surgeons in Israel raised their concerns about performing elective BS during the acute phase of the pandemic. On the patients side, morbidly obese patients are a vulnerable group with higher incidence of severe comorbidities (diabetes, hypertension, respiratory conditions, ischemic heart disease to mention a few) than the general population. Because of strict measures of isolation, social distancing and even lockdown implemented by the government, the post-operative course once the patient is discharged to his/her community was also uncertain. Additionally, bariatric surgery with its potential early post-operative complications challenges the immune system and requires an appropriate response. Obesity is associated with metabolic disturbances that cause tissue stress and dysfunction. The physiological dysfunction that underlies obesity leads to fat accumulation in primary lymphoid organs (bone marrow and thymus) [13, 14]. These changes lead to alterations in the distribution of leukocyte populations, lymphocyte activity, and overall immune defenses [13, 15–17]. Despite having increased basal levels of inflammatory leukocytes, obesity is associated with impaired immune responses and increased mortality in sepsis) [18)].

Thus, the gut feeling was that in certain circumstances the outcome of elective BS may be detrimental. This feeling was supported by an early report by Aminian et al. [19] describing an unexpected fatality in a morbidly obese patient planned to undergo RYGB. The patient developed severe respiratory distress and died a day before the scheduled operation. In a later review of the literature, Nahshon et al. [20] reported a 27.5% postoperative mortality rate and severe complications in patients diagnosed post-operatively as COVID 19 carriers. The authors recommended considering pre-operative testing to all elective surgeries, a practice not in use in Israel at that time. Indeed, during March 2020 the IFSO leadership, using social media platforms (https://www.facebook.co/groups/ifso.global/permalink/2628606017262717/), and the American College of Surgeons [21, 22] advised against performing elective surgery at that time. The IFSO reimplemented those recommendation in a recent publication [23] as did Rubino et al. In a personal view article [24].

On the medical staff’s side, the concerns regarded the potential for exposure and transmission of the virus bilaterally. Surgery in a patient carrying the virus may increase risk of infection among caregivers team. During intubation due to the proximity of the anesthesiologist to the patient’s airways droplet infection is likely to happen. In laparoscopic surgery, the abdomen is inflated by carbon dioxide which can spread to the environment. Gas evacuation through trocars and the use of various energy sources, may increase the risk for staff infection as well [25, 26]. In our survey we demonstrate that while 85% of the surgeons in the public sector stopped absolutely performing elective BS, only 61% did the same in the private sector despite all the above theoretical concerns. The majority (77%) of surgeons (in both sectors) who continued to operate during the pandemic did not apply any special “new” contra-indications to surgery, and only half of them informed their patients about increased perioperative risk. The 24% of the surgeons who adopted more strict preoperative criteria referred to age (over 60y or over 70y), background morbidity (diabetes, hypertension, ischemic heart disease, chronic lung disease) and whether the patient came from a highly infected geographical area.

### Resuming elective BS

Surprisingly, when asked on future conditions needed to resume elective BS, the most popular reply was that no special measure are needed. Asking for a negative PCR COVID-19 test (27%) and a positive IgG (15%) as conditions for performing elective BS in the future were popular options as well, but lower than expected. This can be interpreted as regarding patients and staff safety by many of the responders as a minor issue when deciding when and how to resume elective BS.

Several questions remain open after this survey. Why did not all the administrators in the public sector comply with the MOH decree? Why did some surgeons (14%) continue to perform elective BS against the advice of professional associations and societies [15–17]? Was it right by the MOH to differentiate the regulation between sectors? Was not such a decision a major contributor to the loose way it was implemented by administrations and surgeons alike? Why did not the MOH enforce its own decree? At the time being, the answers to those questions are merely speculative. Nevertheless, our opinion is that one needs extremely specific and crystal-clear, non-ambiguous, policies that impose sanctions on the institutions and/or their staff for violation in a situation like the current pandemic. On the other hand, maybe a clear statement by the government that hospitals and/or surgeons will be compensated for elective surgery losses, would increase administrators and/or surgeons compliance. More in-depth research is needed to clarify those issues for an optimal response to the next waves of COVID-19 or other pandemics in the future.

The strength of this survey lies in its nation-wide character and the high response rate (84%) of the surgical community. Social, economic, political, and medical conditions vary greatly between countries, therefor causing, substantial differences in countries’ response to the pandemic [27]. Pooling together data about real life practices and surgeons attitudes from many countries may overlook those differences and skew conclusions and future recommendations. We acknowledge few limitations to our survey. First, this is a web-based survey and despite a high response rate, those participating may be a selective group. Second, surgeons were not asked to the reasons of the decline in operations. It might well be that part of the decline reflects patients preferences and not surgeons’ attitude to the pandemic. Third, the survey did not address directly hospital administrations so their attitudes can be only assumed from the surgeons’ responses.

## Conclusion

lack of scientific information at the beginning of the pandemic, conflicting interests of the parties involved, apparent lack of equality between the public and private sectors and lack of enforcement by the regulator led to very diverse practices and attitudes among bariatric surgeons in Israel. We call for a more coherent and transparent decision making process by all parties in the future. With respect to practical recommendations concerning bariatric surgery in times of COVID-19 pandemic, since obesity is considered a risk factor for infection, especially with severe clinical course, and since it is clear by now that the COVID-19 pandemic will accompany us for a while, considering that vaccinations are known to be less effective in obese patients ([28]), our recommendation is to continue to perform bariatric surgeries only to PCR negative patients and to place patients postoperatively in quarantine for two weeks (the period with the highest risk of postoperative complications).

## Data Availability

All data generated or analysed during this study are included in this published article. The datasets are available from the corresponding author on reasonable request.

## Abbreviations

BS: Bariatric surgery
MOH: Ministry of Health
OR: Operating room
IFSO: International Federation for the Surgery of Obesity and Metabolic Disorders
BMI: Body mass index
ICU: Intensive care unit
ARDS: Acute respiratory distress syndrome
IRB: Institutional review board
PPE: Personal protective equipment
HMOs: Health Medical Organizations
RYGB: Roux-en-Y Gastric Bypass
